# Supplementary material for: Discrete spatial organization of TGFβ receptors couples receptor multimerization and signaling to cellular tension
Source: eLife. 2015 Dec 10;4:e09300. doi: 10.7554/eLife.09300 (PMC4728123; doi:10.7554/eLife.09300)
Supplement: Figure 2—source data 1. — DOI: http://dx.doi.org/10.7554/eLife.09300.006 [file elife-09300-fig2-data1.zip › Figure 2 - Source data/Readme.pdf]

This zip contains representative sptPALM imaging sequences of mEos2-labeled TβRI and TβRII in ATDC5 cells (50 frames, 10 frames/sec) and their corresponding tracking analysis outputs (track number, frame, x and y coordinates).
